# More than a token photo: humanizing scientists enhances student engagement

**DOI:** 10.1098/rspb.2024.0879

**Published:** 2025-01-22

**Authors:** Robin A. Costello, Emily P. Driessen, Melissa K. Kjelvik, Elizabeth H. Schultheis, Rachel M. Youngblood, Ash T. Zemenick, Marjorie G. Weber, Cissy J. Ballen

**Affiliations:** ^1^ Department of Biological Sciences, Auburn University, Auburn, AL 36849, USA; ^2^ Department of Biological Sciences, University at Buffalo, Buffalo, NY 14260, USA; ^3^ Department of Biology Teaching and Learning, University of Minnesota, Minnesota, MN 55455, USA; ^4^ Department of Plant Biology, Michigan State University, East Lansing, MI 48824, USA; ^5^ Kellogg Biological Station, Michigan State University, Hickory Corners, MI 49060, USA; ^6^ Department of Ecology and Evolutionary Biology, University of Michigan, Ann Arbor, MI 48104, USA

**Keywords:** biology undergraduate education, counter-stereotypical scientist, equity, relatability, scientist role model

## Abstract

Despite broad consensus that highlighting counter-stereotypical scientist role models in educational materials promotes equity and success, the specific elements that make these materials effective remain untested. Are pictures of counter-stereotypical scientists enough to communicate to students that scientists come from a variety of backgrounds, or is additional information required? To parse the effects of including visual depictions and humanizing information about scientists featured in biology course materials, we distributed three randomized versions of assignments over several academic terms across 36 undergraduate institutions (*n* > 3700 students). We found that including humanizing information about scientists was key to increasing student engagement with the biology course materials. The positive effect of humanizing information was especially important for students who related to the scientists. Structural equation modelling revealed the extent to which students related to scientists mediated the positive effect of humanizing descriptions on student engagement. Furthermore, our results were strongest among students who shared one or more excluded identity(s) with the featured scientists. Our findings underscore the importance of providing students with examples of humanized and relatable scientists in classrooms, rather than simply adding a photo to increase representation.

## 1. Introduction

Undergraduate students in science, technology, engineering, and mathematics (STEM) are unlikely to see a diversity of scientists represented in their educational materials [1–5]. The absence of scientists who possess diverse identities is due to persistent systems of oppression that exclude and marginalize people and that acknowledge a narrow definition of success in science as belonging to elite, primarily white institutions [6–8]. The mismatch between the identities of scientists featured in courses and an increasingly diversified student body [9] upholds the stereotype of scientists as white, cis-men, perpetuating the false narrative that only certain types of people contribute to the scientific enterprise [10–13].

Educators and curriculum developers have worked to combat this false narrative by creating educational materials that highlight scientists from a diversity of backgrounds, intentionally featuring scientists whose identities do not match the dominant stereotype of a scientist (hereafter, counter-stereotypical scientists) [14–18]. Featuring counter-stereotypical scientists in course materials is billed as a scalable, accessible and easy-to-implement tool that increases the recruitment and retention of students with identities historically and currently excluded from STEM [14]. Recent efforts to evaluate these course materials in undergraduate biology courses have documented numerous benefits to students, including positive impacts on students’ science identity, perception of who can participate in science, engagement with biology materials, and course grades [15–20]. While evidence has demonstrated the efficacy of biology classroom interventions that feature counter-stereotypical scientists [15–20], the specific elements of these interventions that are most impactful remain unclear [21,22]. The lack of research in this area leaves educators and curriculum developers without data-supported guidelines for how to effectively develop and employ classroom materials that feature counter-stereotypical scientists.

Theory predicts that counter-stereotypical scientists must be *relatable* for students to engage with STEM content and pursue STEM careers [23]. Students are hypothesized to relate to scientists along two different axes: shared visual elements (i.e. that scientist looks like me) and shared humanizing elements (i.e. that scientist shares similar values, hobbies, and experiences with me) [23]. Humanizing information consists of biographical elements that provide students with a broader, more nuanced and holistic perspective on successful scientists, who may otherwise be considered unrelatable and their success unattainable [24,25]. Here, we test this theory by asking whether a photo is enough to make featured scientists relatable or whether humanizing elements are necessary. Specifically, we employed a large-scale experimental approach in undergraduate biology courses across the United States to parse the effects of visual and humanizing elements of classroom materials featuring counter-stereotypical scientists on how students relate to scientists and engage with biology content.

**Figure 1.**
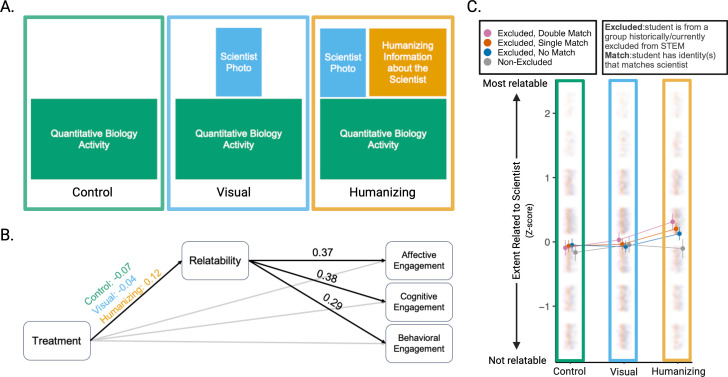
Effects of visual and humanizing elements on how students relate to scientists and engage with quantitative biology activities. (A) Three treatments used in this study varied the extent to which visual and humanizing elements were provided during quantitative biology activities. (B) Our path model found that the perceived relatability of the scientist mediated the relationship between the three treatments and all three dimensions of student engagement on the quantitative biology activities. In particular, the humanizing treatment led to higher scientist relatability which increased student engagement. Black paths are significant (*p* < 0.05). Marginal means are reported for the path connecting treatment to scientist relatability, and mean-variance standardized beta coefficients are reported for paths connecting scientist relatability to the three components of engagement. (C) Marginal means with 95% confidence error bars of the extent to which students related to the featured scientist by treatment and the extent to which the student gender and race/ethnicity identities matched the identities of the scientist featured. Jittered points (*n* = 6244 survey responses from 3103 students) depict the extent to which students related to the scientist on a 7-point sliding scale, ranging from 1 (not at all) to 7 (very), that has been mean and variance standardized.

Prior experimental work on biology course materials featuring counter-stereotypical scientists has been limited to single institutions [15–17,19,20]. To account for differences in social and environmental contexts across universities [26], we conducted a nationwide, multi-institutional study across 36 undergraduate institutions, reaching over 3700 students. We experimentally manipulated the way biology activities highlight counter-stereotypical scientists. Students were given one of three versions of short quantitative exercises (Data Nuggets [27,28]) that feature data from contemporary scientists who self-report as having identities excluded from biology (Project Biodiversify [29]). As treatments, students were exposed to: (i) quantitative exercises that do not include any information about the scientist aside from their name and pronouns (control); (ii) quantitative exercises with photos of the scientist (visual treatment); or (iii) quantitative exercises with scientist photos and interview questions answered by the scientist about their experiences as counter-stereotypical scientists (humanizing treatment) (figure 1A).

We used this experimental design to investigate the effects of visual and humanizing elements of counter-stereotypical scientists featured in biology course materials. Specifically, we address three research questions.

1. *What enhances student engagement?* We tested which features of counter-stereotypical scientists are responsible for creating change in how students engage with biology materials. We further employed structural equation modelling (SEM) to explicitly test theoretical predictions that the extent to which students relate to scientists drives student engagement with course content [23].
2. *Does it matter whether students share the same identities as the featured scientists?* Exposure to curricular materials that feature counter-stereotypical scientists is expected to shift attitudes towards science and scientists, especially among students who share identities with the featured scientists [21,22]. To test this hypothesis, we demographically matched counter-stereotypical identities of featured scientists and students.
3. *What exactly are students relating to?* Finally, we analysed qualitative student responses to better understand which elements of the featured scientists were most relatable to students.

Answers to these research questions will inform the development of biology curricular materials that ultimately improve equity in undergraduate biology education.

## 2. Material and methods

### (a) Experimental design

We used a multi-level experimental design in which instructors of undergraduate biology courses in the United States applied three randomized treatments across academic terms (figure 1A). The three treatments were designed to assess the impacts of quantitative biology activities that varied in the extent to which they included information about featured counter-stereotypical scientists. Each academic term involved the implementation of three activities from a single treatment. Activities emphasized data interpretation from research conducted by the featured scientists (e.g. identifying hypotheses, making predictions, and drawing graphs from data), and the assignments were embedded throughout the academic term. Our materials were written based on the science and lived experiences of 12 biologists who self-reported possessing excluded backgrounds and identities (electronic supplementary material, table S1).

### (b) Material development

We developed classroom materials that pair profiles of counter-stereotypical scientists with quantitative biology exercises for use in this study. These activities were created by combining elements of two pre-developed programs: Data Nuggets activities (quantitative biology activities that use authentic data from real researchers in K−16 classrooms, https://datanuggets.org/) and Project Biodiversify Scientist Profiles (researcher profiles of counter-stereotypical scientists for easy use in classrooms, https://projectbiodiversify.org/). We invited scientists to participate in our study who had already created either a Data Nugget activity or a Project Biodiversify Scientist Profile. We created course materials in close colloabroation with the featured scienitsts, placing priority on the development of content that reflects the scientists' stories. All scientists were offered compensation for their work developing these course materials. All materials used in this study are freely available for download at https://datanuggets.org/dataversify/. See the electronic supplementary materials for an example classroom activity.

Each activity included three elements when presented in full: (i) a brief overview of a scientific study with associated quantitative biology questions, (ii) photos of the scientist who conducted the study, and (iii) humanizing information about the scientist. Data Nuggets feature scientists and provide real data and images from their research, alongside scientific background information, a graphing activity, and questions about the study for students to answer. Project Biodiversify scientist profiles include both visual and humanizing elements about the scientist. Visual elements consist of two photos of the scientist—one at work and one from life outside of science, both chosen by the scientist. Examples of photos included the scientist spending time with their family, enjoying nature, and participating in Comic-Con. Project Biodiversify profiles also allow scientists to share humanizing information revealed through open-ended response prompts answered via google forms by the scientist about their life and science experiences. In the Project Biodiversify profiles, scientists responded to the following questions:

1. Why did you become a biologist?
2. What is your favourite part about your job?
3. What obstacles have you overcome to get where you are?
4. What advice do you have for aspiring biologists?
5. Do you feel that any dimension of your identity is invisible or under-represented/marginalized in STEM?
6. Can you elaborate on your answer above?

Examples of scientists’ experiences shared in their profiles include discussions of challenges learning English, education quality gaps, discrimination faced during gender transitions, and difficulties associated with mental health. See the electronic supplementary material for an example of a full scientist profile.

### (c) Treatments

Our treatments manipulated which elements of the activities were presented in classrooms across three academic terms. This approach allowed us to use the same core content and quantitative biology instruction, while varying the extent to which we shared scientific visual and humanizing content (figure 1A). The control treatment exposed students to Data Nuggets, but photos and additional information from the Project Biodiversify scientist profiles were not included. In the visual treatment, students were exposed to Data Nuggets as well as one image of the scientist. In the humanizing treatment, students were exposed to Data Nuggets and the scientist profiles from Project Biodiversify (i.e. multiple scientist photos and humanizing information). Of note, our control treatment incorporated scientists’ names and pronouns, which can communicate information about gender and race/ethnicity identities. As such, the comparison between the control and visual treatments evaluates the impact of visual depictions of scientists, beyond names and pronouns.

### (d) Instructor recruitment

We recruited 43 instructors from 36 US academic institutions across 20 states (electronic supplementary material, table S2). To recruit instructors, we used Data Nuggets and Project Biodiversify mailing lists and social media accounts, suggestions from members of our network, academic and conference listservs, and the NSF Research Coordination Network project, Equity and Diversity in Undergraduate STEM (NSF-RCN-UBE-1919462, 1 September 2019 to 31 August 2023). We selected individuals from 289 applicants after reviewing their course schedules and considering the best fit for our study design. In total, 126 instructors met the parameters of our study, and 43 instructors accepted our invitation to participate in the study. During the study, instructors implemented materials in 37 introductory-level biology courses and four upper-level courses. Instructor participants represented a broad population who taught at R1 universities, liberal arts institutions, primarily undergraduate institutions, and community colleges. Of the 32 instructors who provided demographic identities, 27 were women and 5 were men. This subset also included 29 white/European American, 2 Asian/Asian American, 2 Black/African American and 1 Latino/a/x/Hispanic American instructors. Two of these instructors identified with multiple race/ethnicities. All instructors were compensated for their participation in the study.

### (e) Content matching and featured counter-stereotypical scientist selection

We worked directly with each instructor to select three out of 12 quantitative biology activities that best matched their course content, while still highlighting a diversity of counter-stereotypical scientists. Our aim was to centre scientists with excluded identities, and, for this reason, we do not have a treatment that features white cis-man scientists, as they are not an excluded demographic group in biology. To ensure each student in our study saw similar levels of representation, we required that each instructor included (i) a scientist who identifies as a minoritized gender (e.g. a woman, or transgender or gender non-conforming person) and (ii) a scientist of colour. We chose these two categories because they are both considered to be excluded groups in biology and, in contrast to concealable stigmatized identities, can often be, but not always, visually identified (important for the visual treatment). However, we recognize that many gender and racial/ethnic identities are concealable and note that several featured scientists in our study revealed their identities in the humanizing, and not visual, treatment. For example, one of our featured scientists identified as transgender and nonbinary (electronic supplementary material, table S1), and they discussed in their scientist profile the serious challenges and discrimination that transgender individuals face when pursuing educational and professional goals. Another scientist identified as both white and Latino/a/x (electronic supplementary material, table S1) and discussed their experience as the child of an immigrant from South America in their scientist profile. We further recognize that women are currently awarded the majority of bachelor’s degrees in the biological and biomedical sciences [30]. However, we consider women a minoritized gender in this study, as they still make up a minority of scientists in biology textbooks [5].

### (f) Student demographics and outcome measures

We surveyed students with short, online Qualtrics surveys at the beginning and end of the academic term to collect demographic information, including information about race/ethnicity and gender (electronic supplementary material, table S3). Categories for race/ethnicity were based on standards for the classification of federal data on race/ethnicity, commonly used for federal data collection purposes including the decennial census.

To measure how students related to the featured scientists and engaged with the quantitative biology activities, we also surveyed students immediately after they completed the activities. To quantify scientist relatability and student engagement (including subscales for affective, cognitive, and behavioural engagement), we developed and adapted survey items that used the Experience Sampling Method, a survey approach that asks students to report their thoughts and feelings immediately following exposure to the activity [31,32]. All survey items asked students to respond to the survey questions on a 7-point sliding scale, ranging from 1 (not at all) to 7 (very). We measured the validity of our engagement construct using confirmatory factor analysis (CFA; see next section for further detail) [33]. To understand *how* students related to scientists, we asked students to respond to the open-ended prompt: ‘Describe how you related to the featured scientist in the activity, if at all’. De-identified survey responses are available on Dryad [34].

### (g) Confirmatory factor analysis

Immediately after completing each quantitative biology activity, we surveyed students to measure how students related to the featured scientists and engaged with the activities. Our measure of scientist relatability included one item (if a scientist was mentioned, to what extent did you relate to the featured scientist in the activity?), and our measure of student engagement included seven items, incorporating items for affective (was the activity interesting?, did you enjoy the activity?, how engaging did you find the activity?), cognitive (how important was the activity to you?, how important was this activity to your future?), and behavioural (how hard were you working on the activity?, how well were you concentrating on the activity?) engagement [32]. For our measure of engagement, we specified a three-factor confirmatory factor analysis using the R package *lavaan* [35]. Confirmatory factor analyses evaluate whether survey items are more closely related to items within their intended set (or factor) rather than to items outside of their factor [33]. Each of our three factors included items that corresponded to either the affective (i.e. interest in the activity), cognitive (i.e. perceived importance of the activity), or behavioural (i.e. effort put into the activity) dimension of student engagement, as we expected based on the original instrument. The model fit of our three-factor CFA was good according to Chi-square statistics and common fit indices ($\chi^{2}$ = 708.468, df = 11, *p* < 0.0001; CFI = 0.982; TLI = 0.965; RMSEA = 0.092; SRMR = 0.024). Given the validity of the three engagement factors in our student sample, we used the values of these three latent variables in our CFA model as our measures of affective, cognitive, and behavioural engagement.

### (h) Statistical analyses

We used path models and mixed models to explore the effects of visual and humanizing elements of featured scientists on how students related to scientists and engaged with biology activities.

#### (i) Mean-variance standardization

In all of our models, we mean-variance standardized our measures of scientist relatability and student engagement. To mean-variance standardize, we calculated Z-scores using the formula Z-score = (X − μ) / s, where X is either the scientist relatability score or the engagement value measured from the CFA model, μ is the mean relatability or engagement value across the entire student sample, and s is the standard deviation around the mean. Z-scores reflect how many standard deviations a student’s score is from the average of all students across all undergraduate institutions in the sample.

#### (ii) Random effects

Furthermore, as classroom contexts have the potential to modulate the impacts of curricular interventions, all of our models included both the course and the specific activity implemented as random effects [36]. We also included student ID nested within the course to account for repeated measures and avoid pseudoreplication.

#### (iii) What enhances student engagement?

We employed piecewise SEM of path models to examine whether student engagement with the activities was mediated by how relatable students found the featured scientists across different levels of exposure to the featured scientist (i.e. control, visual, or humanizing treatments) [37]. SEM is a statistical approach that explores the relationships between several variables simultaneously using a set of linked regression equations and tests the mediating pathways through which one variable influences another variable [38]. Traditional path analysis using SEM assumes normality and independence of observations. To account for repeated measures from each student over an academic term, we employed piecewise SEM of path models using the R package *piecewiseSEM* [37,39].

We evaluated two separate models to explain each of the components of student engagement (affective, cognitive, and behavioural engagement): the full mediation model and the partial mediation model (electronic supplementary material, figure S1). The full mediation model tested whether scientist relatability fully mediated the relationship between treatment and student engagement, whereas the partial mediation model additionally incorporated a direct effect between treatment and engagement. Piecewise SEM uses Fisher’s C statistic to evaluate the effect of missing paths (in this case, the direct path between treatment and engagement in the full mediation model) on the goodness-of-fit of the model [37]. Models fit the data when the paths missing from the model do not differ from zero, indicated by a Fisher’s C statistic P_C_ > 0.05. As all of our full mediation models fit the data (affective engagement: P_C_ = 0.189; cognitive engagement: P_C_ = 0.207; behavioural engagement: P_C_ = 0.812), we then performed chi-square difference tests to evaluate whether the partial mediation models provided better fits than the full mediation models.

#### (iv) Does it matter whether students share the same identities as the featured scientists?

We used linear mixed models to test how sharing counter-stereotypical identities of featured scientists impacted the extent to which students relate to scientists across the three treatments. We created four categories of student–scientist demographic matching with a focus on excluded identities, which we define as non-cis-man genders and non-white races (see electronic supplementary material text for detailed information). If the student did *not* possess an excluded identity (i.e. was a white cis-gender man), that student was included in the non-excluded category. If the student held excluded gender and/or race/ethnicity identity(s), that student either shared both identities (excluded, double match), shared only one identity (excluded, single match), or did not share those identities with the featured counter-stereotypical scientist (excluded, no match).

These categories allowed us to test how sharing counter-stereotypical identities with featured scientists impacts the extent to which students relate to scientists. Our linear mixed model included treatment, demographic matching categories, and the interaction between the two as fixed effects. We fit our models with the R package *lme4* and specified a binomial distribution [40]. To directly compare students with different degrees of shared identities, we performed post-hoc Tukey pairwise comparisons and calculated Cohen’s *d* effect sizes with the R package *emmeans* [41]. Because 4 of the 12 featured scientists were white women (electronic supplementary material, table S1) and 38% of the students in our sample were white women (electronic supplementary material, figure S2), we also ran a sensitivity analysis that excluded data from activities that featured white women scientists (electronic supplementary material text).

#### (v) What exactly are students relating to?

We employed mixed effects logistic regression models to explore the effect of student–scientist demographic matching on how students responded to the open-ended prompt, ‘Describe how you related to the featured scientist in the activity, if at all’. For this analysis, we only included students in the humanizing treatment, as our goal was to understand how students relate to the featured scientists when the activity included humanizing information about the scientists.

For this qualitative data analysis, we first described the types of student responses to the open-ended prompt through inductive coding. We generated codes inductively from a close reading of student responses rather than searching the text of the responses for a predetermined list of categories [42]. To create codes, two researchers independently reviewed the first term of student responses, independently developed categories that characterized student responses (i.e. codes), met to compare and revise the codes, and developed a unified codebook. Our final codebook included 24 different codes (electronic supplementary material, table S4). After the creation of the codebook, the same two researchers used axial coding [42] to group and abstract codes into four categories (electronic supplementary material, table S4).

Researchers used this final codebook to first independently code student responses and then collaboratively reach consensus. Over the course of our research project, five different researchers used the final codebook to code student responses. One co-author led this qualitative coding process, coding every student response and collaboratively reaching consensus with another researcher for every student response; this ensured consistency in how responses were coded. Researchers assigned responses to all appropriate codes, meaning a single response could fit in multiple codes. Each student response was coded by at least two different researchers, and each coder independently coded responses in ‘blocks’ ranging from 20 to 1073 responses. After coding a block of responses, the two researchers met to reach 100% consensus. The average initial percentage agreement for the blocks was 59%.

We integrated these codes into mixed effects logistic regression models to determine whether student–scientist demographic matching affected how the student related to the featured scientists. Specifically, we employed four different models, one for each category of student response, to measure the effect of demographic matching on the likelihood that the student (i) related to the excluded identity(s) held by the feature scientists, (ii) related to the humanizing information provided in the scientist profiles, (iii) related to the research interests of the featured scientists, and (iv) did not relate to the featured scientists. We fit our models with the R package *lme4* and specified a binomial distribution [40]. We included course, student ID nested within course, and quantitative biology activity as random effects in our logistic regression models.

All analyses were performed using R version 4.3.1.

### (i) Positionality statement

We acknowledge that the identities and backgrounds of the authorship team impact the research process (i.e. the questions asked, the data interpretation). Our authorship team shares some identities with the featured counter-stereotypical scientists and student body. Of note, however, all authors on the team are white, which limits our ability to identify with the lived experiences of many students and scientists who have faced barriers on the basis of their race/ethnicity and introduces bias into our study. We counteracted potential biases in the study design, survey development, and data analysis by critically engaging with the materials and questioning each other’s assumptions when analysing and drawing conclusions from the data.

## 3. Results and discussion

### (a) Student population

Our study included 3788 students from undergraduate biology courses for majors and non-majors in the United States. A subset of students provided their complete demographic information (*n* = 3102 students). In this subset, our student sample comprised 1829 white, 334 Asian, 325 Black, 299 multi-racial, 286 Latino/a/x, 20 American Indian or Alaska Native, and 9 Native Hawaiian or other Pacific Islander students. Additionally, when parsing our student sample by gender, 2002 students identified as women, 978 identified as men, and 122 identified as intersex and trans-spectrum (i.e. transgender, nonbinary, and/or genderqueer). Student demographics did not differ across the three treatments (electronic supplementary material, figure S2). The number of students who responded to our survey across all 43 instructors ranged from 11 to 246, with an average of 88 students per instructor.

### (b) What enhances student engagement?

When quantitative biology activities included humanizing information about counter-stereotypical scientists (humanizing treatment), students related more to the scientists (*p* < 0.0001; *n* = 7361 survey responses from 3788 students; partial mediation models; figure 1*b*; Table 1). Students in the humanizing treatment rate the extent to which they relate to the featured scientists by at least 0.16 standard deviations more than students in the visual and control treatments (marginal means: control = −0.074, visual = −0.040, humanizing = 0.124; Table 1). Furthermore, relating more to humanized scientists was associated with higher student engagement with the quantitative biology activities. This was true across three proxy measures for student engagement: (i) interest in the activities (affective engagement; *β* = 0.37; *p* < 0.0001), (ii) perceived importance of the activities (cognitive engagement; *β* = 0.38; *p* < 0.0001), and (iii) effort spent on the activities (behavioural engagement; *β* = 0.29; *p* < 0.0001; figure 1*b*; Table 1).

**Table 1.** Estimates, standard errors (s.e.), and significance values for paths included in the partial path models. Each partial path model included a different component of student engagement: affective, cognitive, and behavioural engagement. Mean-variance standardized estimates are reported for continuous variables and marginal means are reported for categorical variables. Significant *p*-values at the *α* = 0.05 level are bolded.

model	path	estimate	s.e.	*p*‐value
**all**	**treatment to relatability**	**control = −0.074**	**0.037**	**< 0.0001**
		**visual = −0.040**	**0.035**	
		**humanizing = 0.124**	**0.037**	
**affective engagement**	**relatability to affective engagement**	**0.370**	**0.010**	**< 0.0001**
	treatment to affective engagement	control = 0.016	0.051	0.370
		visual = −0.019	0.050	
		humanizing = −0.046	0.051	
**cognitive engagement**	**relatability to cognitive engagement**	**0.378**	**0.010**	**< 0.0001**
	treatment to cognitive engagement	control = 0.029	0.039	0.565
		visual = −0.020	0.038	
		humanizing = −0.023	0.039	
**behavioural engagement**	**relatability to behavioural engagement**	**0.289**	**0.011**	**< 0.0001**
	treatment to behavioural engagement	control = −0.008	0.042	0.480
		visual = −0.013	0.041	
		humanizing = −0.030	0.043	

Partial mediation models provided better fits of the data compared to full mediation models across all three components of engagement (affective engagement: $X_{diff}^{2}$ = 19.875, P_diff_ < 0.0001; cognitive engagement: $X_{diff}^{2}$ = 21.092, P_diff_ < 0.0001; behavioural engagement: $X_{diff}^{2}$ = 16.432, P_diff_ < 0.0001; Table 1). We found support for partial mediation models despite non-significant direct links between treatment and engagement (electronic supplementary material, figure S1; Table 1).

These results illustrate that humanizing information about counter-stereotypical scientists increases the extent to which students relate to those scientists, which in turn translates to higher student engagement. The observed increases in scientist relatability and student engagement align with prior work documenting the impact of incorporating counter-stereotypical scientists into biology curricular materials [15–20]. Our mediation analyses build on this literature base and provide explicit support for theoretical predictions that scientists featured in biology courses must be relatable to increase student engagement with STEM materials [23]. Furthermore, our findings emphasize that simply describing research, mentioning scientists’ names, or including photos of scientists is not enough to increase scientist relatability and student engagement. By presenting counter-stereotypical scientists as ‘normal’ people who face obstacles and hold excluded identities, biology instructors and curriculum developers can create shifts in how students engage with course materials. As student engagement has been repeatedly shown to positively predict academic performance [43–46], incorporating humanizing information about scientists into biology courses can ultimately support student success in STEM.

### (c) Does it matter whether students share the same identities as the featured scientists?

The extent to which students related to the featured scientists depended not only on the information provided about the scientists (treatment: $X_{2,\;6244}^{2}$ = 29.387, *p* < 0.0001; *n* = 6244 survey responses from 3103 students) but also on the extent to which students and featured scientists shared identities systemically excluded from biology (demographic matching: $X_{3,\;6244}^{2}$ = 15.445, *p* = 0.0015). We found that the effect of student–scientist demographic matching varied across our three treatments (treatment × demographic matching: $X_{6,\;6244}^{2}$ = 15.024, *p* = 0.020). Students who shared one or more excluded identity(s) with the featured scientists rated those scientists as most relatable when activities included humanizing information (*double match* vs *non-excluded*: Cohen’s *d* = 0.331, *p* = 0.0005; *single match* vs *non-excluded*: Cohen’s *d* = 0.245, *p* = 0.007; figure 1*c*; Table 2). Conversely, activities that included only visual elements of the featured scientists were not sufficient to create change in how students with shared excluded identity(s) related to the scientists (*double match* vs *non-excluded*: Cohen’s *d* = 0.060, *p* = 0.999; *single match* vs *non-excluded*: Cohen’s *d* = 0.006, *p* = 1.00; figure 1*c*; Table 2). The control treatment likewise found no relationship between shared excluded identity(s) and scientist relatability (*double match* vs *non-excluded*: Cohen’s *d* = 0.057, *p* = 1.00; *single match* vs *non-excluded*: Cohen’s *d* = 0.079, *p* = 0.969; figure 1*c*; Table 2). Unlike for students with excluded identities, treatment did not impact how students with non-excluded identities (white men) related to the featured scientists (*visual* vs *control*: Cohen’s *d* = 0.095, *p* = 0.963; *humanizing* vs *control*: Cohen’s *d* = 0.048, *p* = 1.00; *humanizing* vs *visual*: Cohen’s *d* = −0.047, *p* = 1.00; figure 1*c*).

**Table 2.** Summary of pairwise comparisons between all student–scientist demographic matching categories for each treatment group from the linear mixed model estimating the effect of student–scientist demographic matching on scientist relatability. Differences in estimated marginal means (contrast estimate), standard errors of the estimates (s.e.), Cohen’s *d* effect sizes, and significance values from post-hoc Tukey pairwise comparisons are reported. *p*-values were adjusted using the Tukey method for comparing a family of 12 estimates. Significant *p*-values at the *α* = 0.05 level are bolded.

treatment	contrast	contrast estimate	s.e.	effect size (Cohen’s *d*)	*p*‐value
**humanizing**	double match— single match	0.109	0.066	0.087	0.900
	double match—no match	0.185	0.069	0.147	0.245
	**double match— non-excluded**	**0.416**	**0.093**	**0.331**	**0.0005**
	single match—no match	0.076	0.051	0.061	0.942
	**single match— non-excluded**	**0.307**	**0.080**	**0.245**	**0.007**
	no match—non-excluded	0.231	0.083	0.184	0.189
**visual**	double match - single match	0.067	0.059	0.053	0.993
	double match—no match	0.104	0.061	0.083	0.867
	double match— non-excluded	0.075	0.078	0.060	0.999
	single match—no match	0.037	0.045	0.030	1.000
	single match—non-excluded	0.008	0.068	0.006	1.000
	no match—non-excluded	−0.029	0.070	−0.023	1.000
**control**	double match—single match	−0.028	0.060	−0.022	1.000
	double match—no match	−0.041	0.066	−0.033	1.000
	double match—non-excluded	0.071	0.083	0.057	1.000
	single match—no match	−0.013	0.049	−0.010	1.000
	single match—non-excluded	0.099	0.072	0.079	0.969
	no match—non-excluded	0.112	0.077	0.089	0.950

Our results emphasize that including humanizing information about counter-stereotypical scientists in course materials is necessary for students to relate to scientists with whom they share excluded identities. Humanizing counter-stereotypical scientists in biology course materials may have produced differential outcomes between students with excluded and non-excluded identities in part because the experiences of these students in STEM contexts widely differ. Students with excluded identities currently face many barriers in STEM classrooms, including exclusionary classroom environments [47], limited representation in textbooks [1–5], and experiences of microaggressions [48,49]. These barriers ultimately produce demographic disparities in STEM graduation rates [50,51]. Embracing the humanity of a wide range of counter-stereotypical scientists holding many different excluded identities is one simple act that can help drive change and broaden participation in undergraduate STEM education [14].

### (d) What exactly are students relating to?

**Figure 2.**
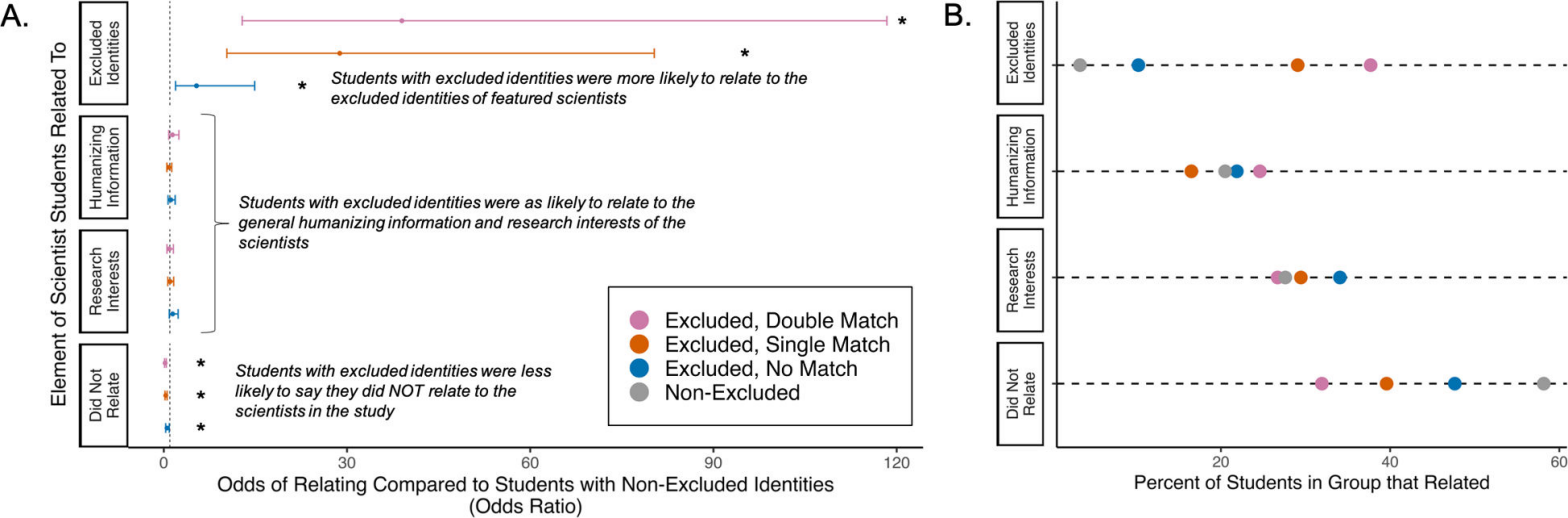
What about scientists did students with systemically excluded identities relate to? (A) Students with excluded identities had higher odds of relating to the excluded identities held by the featured scientists and lower odds of not relating at all to the featured scientists compared to students with non-excluded identities. Odds ratios and 95% confidence intervals of students with excluded identities across each of the four categories characterizing student responses to the open-ended prompt ‘Describe how you related to the featured scientist in the activity, if at all’. The reference group included non-excluded students (i.e. white men). Confidence intervals that do *not* cross the dashed line at *x* = 1 are statistically significant, indicated by asterisks. (B) The percentage of students for each demographic matching category that related to aspects of the featured scientist for each of the four categories.

Qualitative data analysis found that 20.5% of students in the humanizing treatment related to the excluded identity(s) held by the featured scientists, including the scientists’ race/ethnicity, gender, age and sexuality (electronic supplementary material, table S4). Logistic regressions revealed that the extent to which students share excluded identities with the featured scientists impacted the likelihood that students related to the excluded identity(s) of the featured scientist ($X_{3,\;1324}^{2}$ = 59.731, *p *< 0.0001; *n* = 1324 responses from 742 students). Students with shared excluded identity(s) had over 28 times higher odds of reporting relating to the excluded identities of the featured scientists compared to white men students (*double match*: odds ratio = 38.980; *p *< 0.0001; *single match*: odds ratio = 28.807; *p *< 0.0001; figure 2; Table 3). Students with excluded identity(s) but not identity(s) that specifically matched those of the featured scientist still had five times higher odds than white men students of mentioning relating to the excluded identities of the featured scientist (*no match*: odds ratio = 5.354; *p* = 0.0013; figure 2; Table 3).

**Table 3.** Summary of four mixed effect logistic regression models, each exploring the effect of student–scientist demographic matching on how students responded to the open-ended prompt: ‘Describe how you related to the featured scientist in the activity, if at all’. Each model included a different category of student response to the prompt: students either did not relate to the featured scientist, related to the scientist’s research interests, related to humanizing information about the scientist, and/or related to the excluded identity of the scientist. Regression coefficient estimates (estimate), standard errors of the estimates (s.e.), odds ratios, percentages of student responses within each demographic matching category, and significance values are reported. The reference group is non-excluded students (i.e. white men). Significant *p*-values at the *α* = 0.05 level are bolded.

model	fixed effect	estimate	s.e.	odds ratio	**percentage**	*p*‐value
**did not relate**	**double match**	**−1.530**	**0.326**	**0.216**	**31.9%**	**<0.0001**
	**single match**	**−1.129**	**0.263**	**0.323**	**39.6%**	**<0.0001**
	**no match**	**−0.645**	**0.273**	**0.524**	**47.6%**	**0.018**
**relate to research interests**	double match	−0.101	0.284	0.903	26.7%	0.721
	single match	0.032	0.232	1.033	29.5%	0.889
	no match	0.372	0.244	1.450	34.1%	0.128
**relate to humanizing information**	double match	0.349	0.283	1.418	24.6%	0.217
	single match	−0.217	0.240	0.805	16.5%	0.366
	no match	0.134	0.249	1.143	21.9%	0.591
**relate to excluded identities**	**double match**	**3.663**	**0.557**	**38.980**	**37.7%**	**<0.0001**
	**single match**	**3.361**	**0.523**	**28.807**	**29.1%**	**<0.0001**
	**no match**	**1.678**	**0.522**	**5.354**	**10.2%**	**0.0013**

Logistic regressions further revealed that student–scientist demographic matching impacted the likelihood that students did not relate at all to the featured scientist ($X_{3,\;1324}^{2}$ = 27.633, *p* < 0.0001; figure 2). Over half (58.2%) of responses from students with non-excluded identities mentioned not relating to the featured scientists, whereas less than half of responses from double match (31.9%), single match (39.6%) and no match (47.6%) students mentioned not relating at all to the featured scientists (figure 2; Table 3). Finally, student–scientist demographic matching did not impact the likelihood that students related to the research interests ($X_{3,\;1324}^{2}$ = 5.208, *p* = 0.157) or humanizing elements, including hobbies, personality characteristics, and life experiences, ($X_{3,\;1324}^{2}$ = 6.155, *p* = 0.104) of the featured scientists (figure 2). Across all demographic groups, 30.0% and 19.9% of students related to the research interests and hobbies, respectively, of the scientists (figure 2).

Students, regardless of the degree to which they share demographic identities with the featured scientists, related to aspects of the scientist profiles, including the research interests and life experiences of the scientists. This finding emphasizes the impact of brief scientist profiles for students of all backgrounds. However, the scientist profiles used in the humanizing treatment of this study specifically asked scientists to describe dimensions of their identity that are invisible in STEM, and our findings demonstrate that students with excluded identities, especially students who shared those same excluded identities, found the responses to that prompt highly relatable. This analysis reiterates the importance of ‘seeing yourself in science’, as students related most to the identities of the scientists when they shared the same excluded identities as the scientists. However, our results also reveal that students with excluded identities still related to the counter-stereotypical identities held by scientists, even when those students did not share the same excluded identities as the featured scientists. Taken together, this qualitative analysis underscores the importance of providing information about the excluded identities held by counter-stereotypical scientists in biology courses. While previous work has hypothesized the importance of scientists holding meaningfully similar identities, experiences, and interests as students [21,22], this is the first study, to our knowledge, to pinpoint the exact similarities that students most value. Of note, although profiles focused on systemic barriers faced by scientists holding excluded identities, many students related to other aspects of the scientist profiles, like scientists’ research interests, life experiences, personality characteristics, and demographic identities (electronic supplementary material, table S4). Perhaps students were more comfortable naming shared identities rather than discussing obstacles to succeeding in science, a topic that warrants future research.

### (e) Limitations and future directions

The present study has several limitations that will inform future research. First, we applied a quantitative analysis to qualitative student response data, which required reducing student open-ended responses to categorical variables and thereby limited the scope of our interpretation of how students related to the featured counter-stereotypical scientists [52]. Future work will employ qualitative analytical approaches, such as open thematic analysis, to provide a more comprehensive understanding of student perspectives on which elements improve scientist relatability. Second, while our study demonstrates the power of adding humanizing information into classroom materials that feature scientists, it does not address whether other types of information would have larger positive impacts, either alone or in tandem with humanizing information. Finally, we did not evaluate the impact of specific types of humanizing information on student outcomes. While our scientist profiles included the same question prompts, scientist responses varied in both tone and length. Future work will focus on understanding the impact of this variation in the amount and type of humanizing information included in scientist profiles and will help guide content development.

## 4. Concluding remarks

Three key findings emerge from our research. (i) Including humanizing information about the hobbies and experiences of counter-stereotypical scientists featured in curricular materials is necessary for students to relate to scientists. (ii) Students who share one or more excluded identity(s) with humanized scientists relate more than students whose identities do not match featured scientists. (iii) When students relate to scientists featured in curricular materials, they engage more with the materials.

Our results demonstrate that including photos of scientists in course materials is not sufficient to change student attitudes about science. Rather, to engage students, it is critical we also humanize scientists. Exposure to counter-stereotypical scientists is also critically important for students who do not possess excluded identities, to challenge internalized, implicit biases of what types of people can succeed in science [11,53] and reduce the risk they perpetuate a culture of STEM as a white, cisheteropatriarchal space. We recommend several existing resources for use in biology courses, such as DataVersify [27,28] studied here, Project Biodiversify [29], Scientist Spotlights [15–20], BioGraphI, Story Collider Podcast and the SACNAS Biography Project. These materials all vary widely in how they incorporate humanizing information, differing in the amount of information included and the way that information is conveyed (i.e. through text, audio, video). The next step in enhancing curricular resources is to identify the types of humanizing information commonly provided to students and assess their impacts.

Our findings show that it is vital to intentionally centre and humanize counter-stereotypical scientists in curricular materials. By embracing the humanity of scientists, curricular materials can signal to students that they too can contribute to biology. Our work provides a simple approach that curriculum developers and instructional/departmental policies can employ to improve equity in biology.

## Data Availability

De-identified data and code used for data analysis are available at [34]. Curricular materials used in this study are available at [54]. Supplementary material is available online [55].
